# Antifungal Activity of *Artemisia capillaris* Essential Oil Against Alternaria Species Causing Black Spot on Yanbian Pingguoli Pear in China

**DOI:** 10.3390/plants14203146

**Published:** 2025-10-13

**Authors:** Zu-Xin Kou, Yue Dang, Li Liu, Xue-Hong Wu, Yu Fu

**Affiliations:** 1 Department of Chemistry, Yanbian University, Yanji 133002, China; 15841950399@163.com (Z.-X.K.); 13500813498@163.com (Y.D.); 2 Department of Plant Pathology, China Agricultural University, Beijing 100193, China; liulill1124@163.com

**Keywords:** *Artemisia capillaris*, essential oil, Pingguoli pear, antifungal activity

## Abstract

Black spot is currently one of the most widespread diseases affecting Yanbian Pingguoli pears (*Pyrus pyrifolia* cv. ‘Pingguoli’), resulting in significant economic losses for fruit farmers. It is mainly caused by infestation by the fungal group of *Alternaria* species. To date, no research has reported the presence of *Alternaria* species and the pathogen of black spot disease on Yanbian Pingguoli pears in China. This study isolated, identified, and performed molecular profiling of 124 *Alternaria* strains collected from 15 major growing areas of Yanbian Pingguoli pear (more than 5000 trees). Moreover, the study evaluated the ability of *Artemisia capillaris* essential oil (AcEO) to suppress the mycelial expansion of *Alternaria* pathogens and conducted comprehensive chemical profiling. Overall, 124 pathogenic fungi were identified as *Alternaria tenuissima* (67 isolates, 54.0%) and *A. alternate* (57 isolates, 46.0%). AcEO showed a strong inhibitory effect on the two *Alternaria* species, with a minimal inhibitory concentration (MIC) value equivalent to 5.0 μL/mL. Eucalyptol, 2,2-Dimethyl-3-methylenebicyclo [2.2.1] heptane, (-)-alcanfor, and β-copaene were identified as the predominant bioactive components of AcEO. AcEO demonstrated concentration-dependent inhibition of the mycelial growth of *A. tenuissima* and *A. alternata*. These findings position AcEO as a promising candidate for developing sustainable fungicides to combat *Alternaria*-induced crop losses.

## 1. Introduction

Pingguoli pear (*Pyrus pyrifolia* cv. ‘Pingguoli’) belongs to Maloideae of the family Rosaceae. As the pear’s shape is oblate, the background color is yellow and green, the sun gives it a red glow and, from far away, it looks like an apple on the tree, it is called Pingguoli. There are many advantages of the Pingguoli pear, including large fruit, a small nucleus, fine and crisp flesh, high sugar content, fragrant odor, and resistance during storage, making it widely loved by the public [1,2,3]. Yanbian Pingguoli pear, one such excellent pear variety, has been cultivated for more than 100 years in the east of China. At present, its planting scale is 12,000 hectares across more than 10 thousand households and fruit farmers, with an annual output of more than 90 thousand tons, producing an output value of nearly CNY 100 million. Yanbian Pingguoli pear production has become a pillar industry in the east of China, which is Asia’s largest apple and pear production base.

While Pingguoli pears can be stored at cooler temperatures for up to six months, the incidence of black spot is as high as 37% at the later stage of storage [4,5]. Black spot disease is currently one of the most widely observed diseases in Pingguoli pear, and is mainly caused by infestation with the fungal group of *Alternaria* species [6,7], such as Japanese pear [8], European pear [9], Asian pear [10], sandy pear [11] and Korla fragrant pear [12]. It mainly affects the new leaves, new shoots, and young fruits of Pingguoli pear and can invade through the flowers and fruits, causing a large number of diseased fruits and early fruit drop. When the disease is serious, it can even lead to fruit tree defoliation, fruit rot, shedding, and the death of new tips, thus affecting the yield of the fruit tree and resulting in huge economic losses [4,5]. Meanwhile, toxins produced by the pathogenic fungus of black spot on Pingguoli pear can trigger human esophageal cancer development, causing food safety issues [13]. The continuous use of chemical fungicides as the main means of control, such as iprodione, tebuconazole, and mancozeb, has gradually resulted in the increasing resistance of pathogens, reduced sensitivity, environmental pollution, the accumulation of pesticide residues in food and an associated risk of contracting cancer [14]. Therefore, there is an urgent need to develop an effective and environmentally friendly alternative to these traditional fungicides to control black spot on Pingguoli pear.

Plant essential oils, also called volatile oils, are oily liquids containing a variety of volatile organic compounds extracted from the roots, stems, leaves, flowers, fruits, and other parts of plants [15]. There are more than 3000 kinds of plant essential oils, which play a wide range of roles in insecticidal, anti-inflammatory, anti-oxidant, anti-cancer, and anti-microbial activities [16,17,18]. The chemical profiling of plant essential oils reveals a complex matrix of bioactive constituents, primarily comprising hydrocarbon skeletons (terpenoid frameworks) and their oxygenated functional groups (phenolic aldehydes/esters). These compounds encompass terpenes, alcohols, amines, aldehydes, ethers, oxides, ketones, esters, amides, phenols, and heterocycles, forming intricate combinations [19]. Generally, around 60–70% of the total essential oil constituents comprise two to three major compounds [20]. The composition of secondary metabolites within essential oils is influenced by a multitude of factors, including geography, photoperiod, abiotic and edaphic factors, seasons, microbial diversity, the specific plant part utilized, developmental stage, and the method of extraction [21].

Several studies have reported that various plant essential oils showed high efficiency in controlling *Alternaria* species. Perveen et al. [22] found that the *Ocimum basilicum* L. essential oil successfully constrained the growth and conidia germination of *A. alternata*. The highest concentration of basil essential oil (10%) provided maximum growth reduction (88%) in the fungus. Kamsu et al. [23] tested a Massep (*Ocimum gratissimum* L.) essential oil concentration of 2400 μL/L and found it was effective in inhibiting *A. solani* at a rate of 71.81%. Chen et al. [24] showed a 50.80% reduction in *A. alternata* incidence in inoculated blueberries by day 4 post-*Artemisia argyi* essential oil treatment. *Artemisia capillaris* Thunb. is a traditional medicine with suggested hepatoprotective effects, which belongs to the *Artemisia asteraceae* family [25]. Although previous studies have suggested that AcEO has demonstrated antimicrobial effects [26], limited experimental evidence exists regarding its effect on the *Alternaria* pathogenic fungi of black spot disease. The objective of this study was to evaluate the antifungal potential of AcEO against *Alternaria* pathogens associated with black spot disease on Yabian Pingguoli pears. This is expected to provide a new avenue for the development and utilization of *Artemisia capillaris* as a novel biofungicide.

## 2. Results

### 2.1. Sample Collection and Isolation of Alternaria Isolates

During the period spanning 2020 through 2022, specimens of *Alternaria* were cultured from Yanbian Pingguoli pear leaves displaying distinct black spot lesions. These samples originated from 15 Pingguoli pear cultivation sites, encompassing over 5000 trees (Table 1). Notably, both *A. tenuissima* and *A. alternata* species prevailed across all investigated orchards. *A. tenuissima* isolates were not detected in the fruit farm of Longjing County, and *A. alternata* isolates were not detected in Xicheng Town in Helong County and Liangshui Town in Tumen County.

### 2.2. Morphological Characterization of Alternaria Isolates

**Figure 1:**
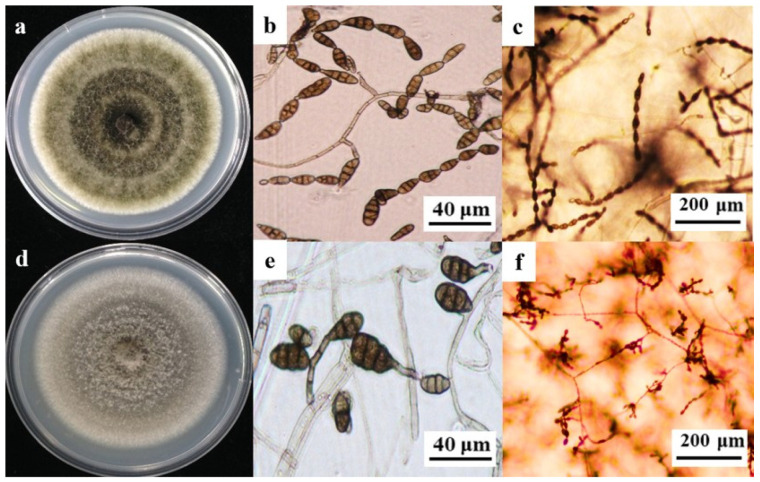
Comparative morphological analysis of *A. tenuissima* and *A. alternata*. (**a**) Colony phenotypes: *A. tenuissima* exhibited tan-colored colonies with radial rugae on PDA. (**b**) Conidial ontogeny: Ovoid conidia ((15.9–31.2) × (5.5–12.2) μm) displaying 1–5 transverse septa. (**c**) Asexual reproductive structures: Linear conidial chains (8–12 conidia per chain) embedded within lateral branches. (**d**) Colony morphometrics: *A. alternata* formed olive-brown colonies with zonate growth patterns. (**e**) Conidial characteristics: Ellipsoidal conidia ((14.8–28.4) × (4.2–10.3) μm) bearing 0–2 intercalary cells. (**f**) Sporulation architecture: Curved conidial chains (5–9 conidia per chain) terminating in phialide clusters.

The morphological analysis of 67 *A. tenuissima* strains revealed initial colony pigmentation ranging from grayish green to olive brown on potato dextrose agar (PDA). Morphometric characterization demonstrated unbranched conidiophores (16.9 μm–54.3 μm in length) terminating in phialides producing conidia (2.9 μm–7.1 μm wide). Conidia exhibited ovoid to obovoid morphology, with dimensions spanning 14.8 μm–28.4 μm (length) and 4.2 μm–10.3 μm (width). On potato carrot agar (PCA), conidial chains displayed 12 conidia per linear arrangement, featuring 1–4 transverse septa and 0–2 intercalary cells, with the entire structure embedded within lateral branches (Figure 1a–c).

The morphological characterization of 57 *A. alternata* isolates revealed initial colony pigmentation transitioning from tan to dark brown on potato dextrose agar (PDA). The pathogen exhibited filamentous conidial chains containing 8–12 conidia per linear arrangement. Conidiophores measured (10.3 μm–48.1 μm (length)) × (4.9 μm–13.4 μm (width)), terminating in phialides that produced ovoid to ellipsoidal conidia ((15.9–31.2) μm × (5.5–12.2) μm). Conidial structures displayed 1–5 transverse septa and 0–2 longitudinal septa, with intercalary cells embedded within septate conidia (Figure 1d–f). These phenotypic traits, including colony morphology, conidial dimensions, and septation patterns, were morphologically congruent with *A. tenuissima* and *A. alternata* reference strains as documented by Simmons [27].

### 2.3. Molecular Characterization of Alternaria Isolates

**Figure 2:**
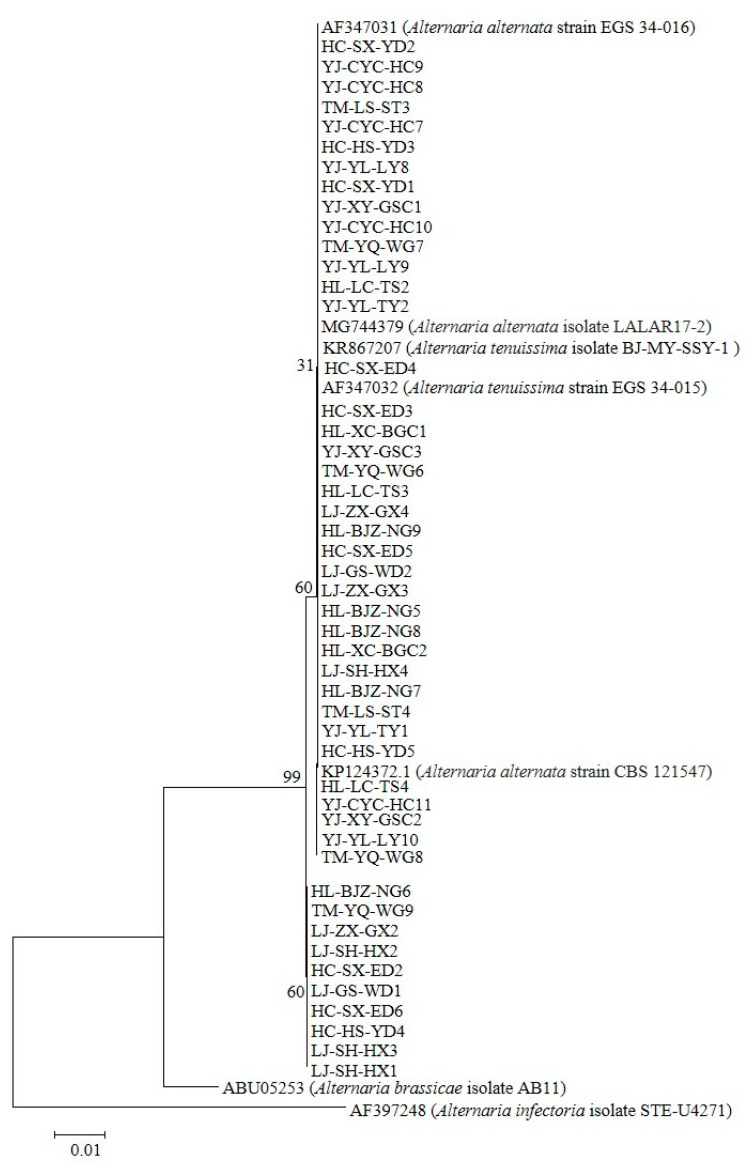
Phylogenetic reconstruction was performed using 48 *Alternaria* pathogenic ITS sequences curated from the National Center for Biotechnology Information (NCBI) nucleotide database. Maximum likelihood analysis generated a topology supported by 1000 bootstrap replicates. The tree was rooted with *A. brassicae* AB11 (GenBank: ABU05253) and *A. infectoria* STE-U4271 (GenBank: AF397248), two phylogenetically distinct taxa representing distinct host adaptation lineages.

**Figure 3:**
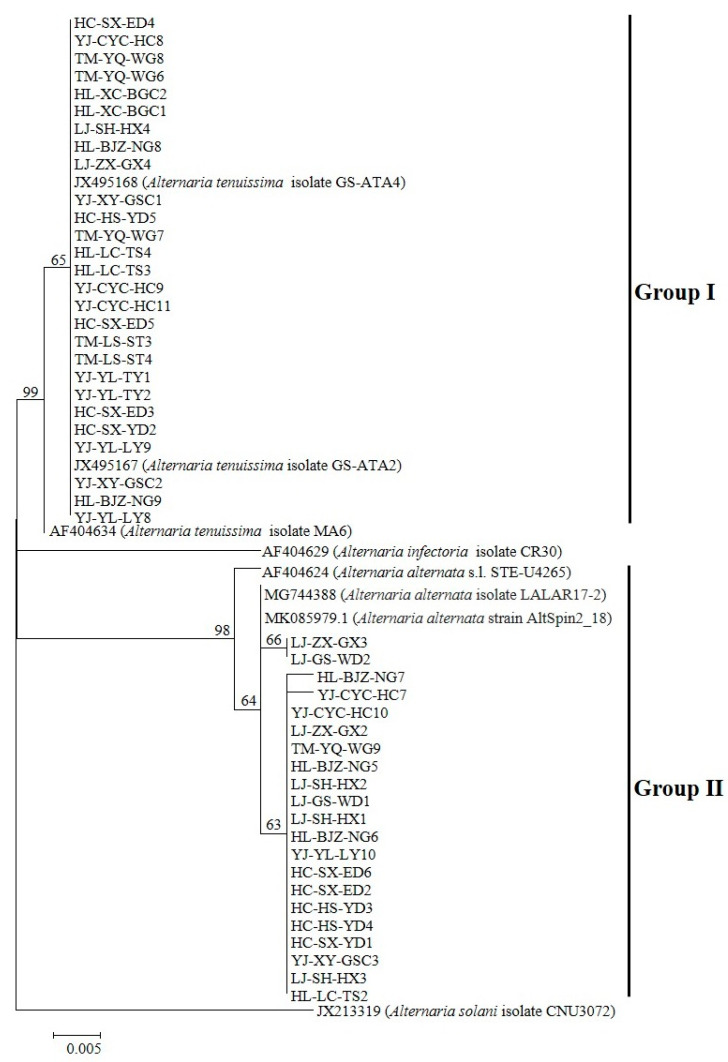
Phylogenetic reconstruction of histone H3 gene sequences (546 bp) resolved 48 *Alternaria* pathogenic strains into two major clades. Bootstrap support (1000 replicates) and Bayesian posterior probabilities (2M generations) demonstrated (1) Group I (*A. tenuissima*) with ≥99% support and (2) Group II (*A. alternata*) with ≥98% support. Outgroup taxa (*A. solani* isolate CNU3072 (JX213319) and *A. infectoria* isolate CR30 (AF404634)) were chosen to root the tree at the *Alternaria* crown group.

The molecular identification of 124 *Alternaria* isolates was conducted via PCR amplification of the ITS region (570 bp) using consensus primers ITS1/ITS4. BLASTn analysis revealed ≥99% nucleotide homology with *A. tenuissima* (KR867207, AF347032) and *A. alternata* (AF347031, MG744379, KP124372.1), along with high similarity to other *Alternaria* spp. in GenBank. Phylogenetic reconstruction based on histone H3 gene sequences (546 bp for Group I; 440 bp for Group II) demonstrated clustering patterns: Group I (*n* = 67) exhibited ≥99% identity to *A. tenuissima* reference sequences (JX495167, JX495168, AF404634), and Group II (*n* = 57) showed ≥98% homology with *A. alternata* strains (AF404624, MG744388, MK085979.1). The complete nucleotide datasets (*n* = 124) have been deposited in GenBank (Supplementary Table S1). Unrooted phylogenetic trees constructed from ITS and histone H3 data (Figure 2 and Figure 3) revealed distinct clades corresponding to the two genotypic groups identified in pathogenicity trials (*n* = 48 isolates).

### 2.4. Pathogenicity of Representative Alternaria Isolates

**Figure 4:**
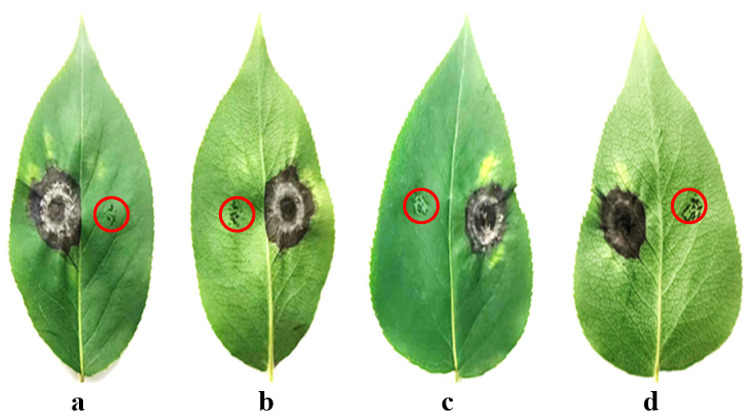
Pathogenicity of 48 *Alternaria* isolates on detached and healthy Yanbian Pingguoli pear leaves (6 cm long and 3.5 cm wide) naturally grown in Pingguoli pear orchards at the end of May. (**a**): Adaxial side of the leaf inoculated with *A. tenuissima*. (**b**): Abaxial side of the leaf inoculated with *A. tenuissima*; (**c**): Adaxial side of the leaf inoculated with *A. alternata*. (**d**)**:** Abaxial side of the leaf inoculated with *A. alternata*.

Following the administration of needle puncture wounds to both the left and right sides of a healthy leaf, the left side was co-inoculated with *Alternaria* isolates. The right puncture site was maintained as a mock-inoculated control, as shown in the areas indicated by the red circles in Figure 4. The manifestation of conspicuous necrotic lesions was observed at five days post-treatment. After two weeks, when Yanbian Pingguoli pear leaves were challenged with *Alternaria* isolates, excised tissues exhibited distinct concentric ring patterns and necrotic spots (Figure 4). Pathogen recovery and molecular identification from symptomatic tissues further validated Koch’s postulates. The pathogenicity assay demonstrated that *A. tenuissima* induced higher disease incidence (51.7–100.0%) and severity (30.5–50.4) on detached pear leaves compared to *A. alternata* (31.2–100.0% and 18.2–29.5). The differences in disease parameters between the two fungi were statistically significant (*p* < 0.05) (Table 2).

### 2.5. Chemical Composition of AcEO Determined by Gas Chromatography–Mass Spectrometry (GC-MS)

The chemical compositions of the AcEOs were determined using GC-MS chromatographic analyses, in which 40 volatile compounds were identified (Table 3). The structural formulas of various components in AcEO are shown in Supplementary Table S2. The spectral identification (>99% confidence) was conducted through the comparison of experimental mass spectra with the NIST mass spectral library (version 20), complemented by chromatographic co-elution validation using reference standards synthesized in-house. The primary chemical constituents of the AcEO predominantly included eucalyptol (60.40%), 2,2-Dimethyl-3-methylenebicyclo [2.2.1] heptane (5.03%), (-)-alcanfor (3.86%), and β-copaene (3.25%).

### 2.6. Antifungal Activity of the AcEO Against Alternaria spp.

**Figure 5:**
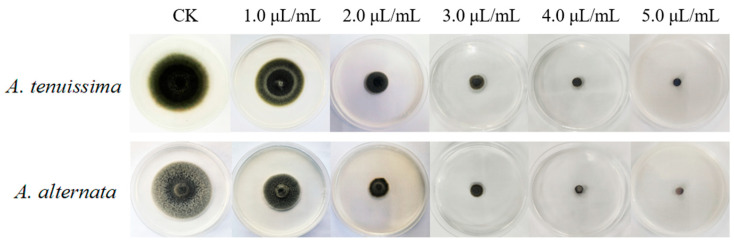
Antifungal inhibitory effects of AcEO on mycelial growth of *A. tenuissima* YJ-XY-GSC1 and *A. alternata* YJ-XY-GSC3 in PDA medium supplemented with gradient concentrations of AcEO (0, 1.0, 2.0, 3.0, 4.0, and 5.0 μL/mL).

As illustrated in Figure 5, incubation at 28 °C for 7 days resulted in the complete colony colonization of *A. tenuissima* YJ-XY-GSC1 and *A. alternata* YJ-XY-GSC3 on agar plates, exhibiting characteristic black flocculent mycelial morphology. Exposure to AcEO induced progressive radial constriction of fungal colonies, with hyphal extension rates demonstrating statistically significant attenuation compared to untreated controls (*p* < 0.01). Dose–response analysis revealed concentration-dependent suppression kinetics, where complete growth inhibition (MIC = 5.0 μL/mL) was achieved through carvacrol-mediated mechanisms. This concentration-dependent inhibition pattern suggests AcEO’s potential as a natural fungicide against these phytopathogens.

**Figure 6:**
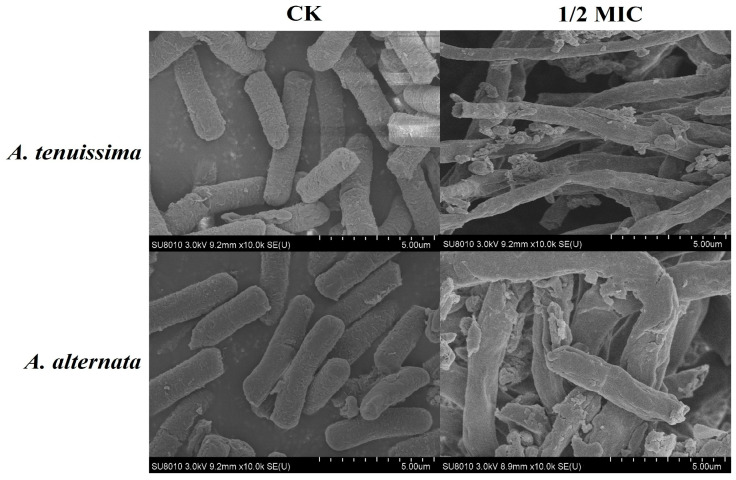
SEM pictures of *A. tenuissima* YJ-XY-GSC1 and *A. alternata* YJ-XY-GSC3 treated with varying AcEO concentrations (0 and 1/2MIC).

Scanning electron microscopy (SEM) analysis revealed dose-dependent ultrastructural modifications in *Alternaria* spp. hyphae. As depicted in the micrographs (Figure 6), radial constriction and surface corrugation became pronounced in *A. tenuissima* YJ-XY-GSC1 and *A. alternata* YJ-XY-GSC3 under AcEO gradients (0.5–5.0 μL/mL), with progressive loss of cytoplasmic continuity at 2.5 μL/mL (1/2 MIC). The control groups (*A. tenuissima* YJ-XY-GSC1 and *A. alternata* YJ-XY-GSC3) displayed intact hyphal networks with uniform cylindrical geometry. The groups with 1/2 MIC of AcEO exhibited pronounced wrinkles and concavity. Notably, this study corroborated emerging evidence that plant-derived essential oils disrupt fungal integrity through the damaged fungal morphology, as previously reported [24,28].

## 3. Discussion

Pingguoli pear was introduced to Yanbian Korean autonomous prefecture in Jilin province in China in 1921 by the Korean cultivator Fandou Cui, and originates from North Korea. After several generations of meticulous cultivation and continuous breeding, it was officially named ‘Pingguoli pear’ in 1958. Today, this fruit is mainly produced in the Yanbian Korean autonomous prefecture in Jilin province, particularly in three counties; namely, Longjing, Helong, and Yanji. Additionally, there are some distribution centers in Tumen, Hunchun, and Wangqing counties, and in other cities. From 1997 to 2004, due to extensive cultivation across provinces or autonomous regions such as Liaoning, Inner Mongolia, and Gansu, Pingguoli pear production surged while prices plummeted into a state of depression. Coupled with limited transportation radius and storage capacity for Pingguoli pears along with increasing production costs over time, fruit farmers witnessed declining profits year after year, leading many to cut down their orchards or repurpose them for alternative uses. Consequently, this resulted in a significant reduction in Pingguoli pear growing areas. Due to the custom of gifting apple pears among Chinese Koreans, the primary growing regions remain within the Yanbian Korean autonomous prefecture of Jilin province, while some are also cultivated in Gansu province. In 2023, Harbin’s tourism boom resulted in a significant increase in the demand for ‘frozen autumn pears.’ These pears are typically obtained from Huagai pear, autumn pear, white pear, and Jianba pear (while the Yanbian area freezes Yanbian Pingguoli pear). Due to their growing popularity, Yanbian Pingguoli pears have attracted more and more attention.

Pear black spot disease, caused by *Alternaria* species, is one of the most serious diseases affecting pear varieties such as Japanese pear [8], European pear [9], Asian pear [10], Korla fragrant pear [29], and sandy pear [11]. Market analysis indicates that Pingguoli pear derivatives are predominantly commercialized through fresh and dried consumption. The pathogenic *Alternaria* strain exhibits robust resistance to cold and is capable of surviving in various environments, including Pingguoli pear trees, leaves, fruits, and soil. Commonly, the Pingguoli pear is storable and can be stored for 6 months at 0 °C, while concurrently exhibiting a high incidence of black spot during the late storage period, reaching up to 37% [30]. This highlights the significant impact of *Alternaria* species on Pingguoli pear production throughout all the storage stages; thus, the fungus has become a pivotal factor impeding the development of the Yanbian Pingguoli pear industry. Previous reports have suggested that *A. alternata* is the dominant etiological factor in black spot disease affecting Pingguoli pears in Gansu province in China [30,31]. To date, no reports have been released announcing that the *Alternaria* species causes black spot on Yanbian Pingguoli pears in China. Therefore, this investigation reveals novel phytopathogenic characteristics of *Alternaria* species on Yanbian pear black spot outbreaks from Jilin Province, China. The molecular characterization of 124 obtained fungi confirms that the selected universal primers ITS1 and ITS4 enabled attribution of the observed disease to *Alternaria*. Furthermore, the partial coding sequence of the histone H3 gene was used to confirm that the *Alternaria* species causing black spot were *A. tenuissima* and *A. alternata*. The phylogenetic analyses in this study are in agreement with other studies, which have shown a clear separation of *A. alternata* and *A. tenuissima* from the *Alternaria* species complex by the partial coding sequence of the histone 3 gene [32,33,34,35]. This study provides the first evidence of *A. tenuissima* and *A. alternata* co-infecting Yanbian Pingguoli, with pathogenic validation through Koch’s postulates and multigene phylogenetic analyses. The observed black spot symptoms, characterized by irregular necrotic lesions with chlorotic halos, mark a novel host–pathogen interaction in China.

*Artemisia* species have been used as food additives and in traditional herbal medicines, particularly for treating diseases such as cancer, inflammation, malaria, hepatitis, and microbial infections [36]. Of these species, *Artemisia capillaris* is known as the Chinese drug ‘Yin Chen Hao’ (‘Injinho’ in Korean medicinal terminology), and is distributed broadly in the north-east area of China. Its traditional effects are to clear away dampness and heat, promote gallbladder function and reduce jaundice [37]. Many investigations have demonstrated that the essential oil of *A. capillaris* exhibits significant biological activity [37]. Our GC-MS findings indicate that the most abundant constituents of AcEO are eucalyptol (60.40%), 2,2-Dimethyl-3-methylenebicyclo [2.2.1] heptane (5.03%), (-)-alcanfor (3.86%), and β-copaene (3.25%). There are significant differences compared to previous reports [38]. The observed phytochemical variability is mediated by environmental parameters including soil mineral composition, solar radiation intensity, growth stage, and nutrient availability gradients.

In this study, we also investigated the effectiveness of AcEO volatiles as a control for *A. alternata* and *A. tenuissima* infestation during Yanbian Pingguoli pear growth. The results showed that the 5.0 μL/mL AcEO treatment completely inhibited the mycelial growth and morphology of *A. alternata* and *A. tenuissima*. Implementing AcEO in Pingguoli commercial practices may decrease chemical fungicide dependency, simultaneously mitigating antifungal resistance development and ecological contamination. The observed phenomena demonstrate synergistic alignment with a study conducted by Chen et al. [24]. The antifungal activity of essential oils (Castor, Jasmine, Clove, Sesame, Neem, Coconut, Henna, Black seed, and Mint) against *A. alternata* was evaluated through radial growth inhibition assays [39]. Extracts were tested at concentrations ranging from 1% to 6% using in vitro inhibition protocols. Singh et al. [28] provided compelling evidence of a significant inhibition of *A. tennussima* growth by *Eucalyptus globulus* essential oil. Various studies have affirmed the antifungal potential of essential oils obtained from a variety of plants on *Alternaria* isolates. Allagui et al. [40] investigated the potential of the essential oil derived from *Cinnamomum verrum* and *Syzygium aromaticum.* Experimental results revealed that these plant-derived extracts exhibited notable efficacy in inhibiting the mycelial growth of *A. alternata*, with MICs ranging from 0.31 to 0.45 mg/mL and 0.37 to 0.57 mg/mL, respectively, as determined through in vitro assays. Despite their proven effectiveness, essential oils face three critical limitations in food system applications: (i) a tendency to volatilize under thermal processing (>40% loss at 60 °C), (ii) aqueous insolubility (<1 mg/mL solubility in PBS), and (iii) chemical instability against oxidative stress (half-life < 24 h). Kamsu et al. [23] confirmed that Massep (*Ocimum gratissimum* L.) essential oil nanoemulsion significantly inhibited the mycelial growth of *A. solani* compared to pure essential oil. Further studies should focus on the preparation of a nanoemulsion using AcEO to maximize the efficacy in controlling pathogens causing black spot in Pingguoli.

## 4. Materials and Methods

### 4.1. Fungal Isolation

The growing area was mainly concentrated in the five cities of Longjing, Helong, Tumen, Hunchun, and Yanji, in Yanbian Korean autonomous prefecture in China. From 2020 to 2022, the pathogenic-infected foliage of Yanbian Pingguoli pear was systematically harvested and then maintained in a 4-degree refrigerated microbial incubator for strain separation. The *Alternaria* isolates causing black spot on Yanbian Pingguoli pear were screened using the single spore separation method, as reported by Fu et al. [35]. Briefly, symptomatic tissues were cleaned with sterile water, disinfected with alcohol and 5% sodium hypochlorite, and cut into small pieces (1–2 mm), which were incubated on potato dextrose agar (PDA) for 7 days. The isolates were selected and purified for subsequent experiments.

### 4.2. Morphological and Molecular Characterization

Purified isolates were measured for conidial morphological parameters, rostrate projection dimensions, morphological silhouette, and transverse septation frequency. The isolates were cultured under controlled conditions including solid medium (potato carrot agar (PCA) poured into 90 mm Petri dishes) and incubation duration (168 h). The morphometric parameters of conidial chains and branching patterns were determined using a Leica M165C stereomicroscope connected to the Image-Pro plus 6.0 image analysis and processing software (Leica, Wetzlar, Germany). The phenotypes of the isolates were systematically evaluated against reference strains of *A. alternata*, *A. tenuissima*, and other species of *Alternaria* [27] through standardized protocols.

Purified isolates were used to extract DNA following the Two-liquid Plant DNA extraction Kit’s protocol (O' BioLab, Beijing, China). PCR amplification targeted the rDNA-ITS fragments with the primer pair ITS1 (5′-TCC GTA GGT GAA CCT GCG G-3′) and ITS4 (5′-TCC TCC GCT TAT TGA TAT GC-3′). The cycling protocol included an initial melting step (94 °C, 5 min), followed by 35 cycles of 94 °C for 40 s (melting), 58 °C for 40 s (annealing), and 72 °C for 1 min (elongation), concluding with a final extension at 72 °C for 10 min. This protocol was optimized to ensure the efficient amplification of conserved regions across diverse fungal taxa [41]. PCR amplification targeted the histone H3 coding region with the primer pair H3-1a (5′-ACT AAG AG ACC GCC CGC AGG-3′) and H3-1b (5′-GCG GGC GAG CTG G1 GTC CTT-3′). The cycling protocol included an initial melting step (96 °C, 2 min), followed by 30 cycles of 96 °C for 15 s (melting), 55 °C for 30 s (annealing), and 75 °C for 35 s (elongation), concluding with a final extension at 72 °C for 2 min. This protocol was designed to maximize yield while minimizing nonspecific amplification artifacts [42]. Sequencing services for the PCR products were conducted through Beijing Tianyi Huiyuan Biotechnology Co., Ltd. Raw sequence data were processed using the DNAMAN bioinformatics software (Version 5.0) to generate consensus sequences, which were subsequently compared against the non-redundant nucleotide database (nt) via the BLASTn algorithm to retrieve homologous sequences from the NCBI library. Maximum likelihood (ML) analysis of concatenated nucleotide datasets from ITS and histone H3 loci was performed to infer phylogenetic relationships among 124 *Alternaria* strains using Clustal_W v1.83 [43] (for multiple sequence alignment) and the MEGA 5 software (release 5.2.2; accessible at http://www.megasoftware.net/) (for tree construction). For the haplotype comparison, phylogenetic analysis of concatenated ITS and histone H3 sequences demonstrated bootstrap support values (1000 replicates) for internal nodes. *A. brassicae isolate* AB11, *A. infectoria* isolate STE-U4271, *A. solani* isolate CNU3072, and *A. infectoria* isolate CR30 were used as outgroups.

### 4.3. Pathogenicity Tests

In order to confirm Koch’s postulates, 48 *Alternaria* isolates with their representation were assessed with relatively minor modifications, as reported by Pryor and Michailides [44]. Briefly, 20 holes were pricked in the recipient leaves with a sterilized prick needle, and a drop of sterile water was placed on one side where the prick was made as a blank. Next, 48 *Alternaria* isolates covered with PDA Petri dishes were pressed out with a hole puncher with a diameter of 5 mm and placed on the side where the holes were pierced with the prick needle. Healthy, detached Yanbian Pingguoli pear leaves (6 cm long and 3.5 cm wide) that naturally grew in Pingguoli pear orchards at the end of May were selected as receptors. Thirty symptomatic leaves were assigned to individual isolation groups and maintained under controlled conditions (25 °C, 90% relative humidity) with a 12 h photoperiod for 14 days. Symptom progression was monitored daily, with diseased tissues subjected to pathogen re-isolation. Disease severity (DS) was quantified using a modified 5-point scale [44]: 0 (no lesions), 1 (<1 mm), 2 (1–5 mm), 3 (5–10 mm), and 4 (>10 mm). The disease index (DI) was calculated as DI = (Σ [*n* × DS])/N × 25, where *n* indicates lesion frequency per grade and N represents total inoculation points [32]. The LSD post hoc analysis revealed statistically significant differences (*p* < 0.05) among experimental conditions.

### 4.4. Extraction of AcEO and GC-MS Analysis

Fresh *Artemisia capillaris* samples were collected from a sunny slope in Yanji City, Jilin Province, China. They were authenticated by an expert from the College of Agriculture, Yanbian University. The whole plants were air-dried in the shade and cut into 5 cm segments. The AcEOs were extracted according to the patent CN117778103B [45]. After 3 h, the AcEOs were collected and stored in brown sample bottles at 4 °C in darkness until further analysis.

GC-MS was performed on a Shimadzu QP 2010 Ultra system (Shimadzu, Tokyo, Japan) equipped with a DB-5MS column (30 m × 0.25 mm ID, 0.25 μm film thickness). The analytical configuration included splitless injection, a helium carrier gas flow (0.98 mL/min), and an injection volume of 2.0 μL. Ionization was achieved via electron impact (70 eV) with the source temperature set at 280 °C. The oven program started at 45 °C (4 min), increased to 280 °C at 6 °C/min, and finalized with a 15 min isothermal hold. Full-scan acquisitions (35–500 *m*/*z*) were acquired at 0.56 scans/s. Compound identification utilized spectral matching against the NIST 20 mass spectral library and was validated through comparative literature analysis [26,46,47].

### 4.5. Antifungal Activity of the AcEO

The antifungal activities of AcEO were tested against the mycotoxigenic strains *A. tenuissima* YJ-XY-GSC1 and *A. alternata* YJ-XY-GSC3, using the contact assay on PDA. For the AcEO, 5 concentrations were used (1.0 μL/mL, 2.0 μL/mL, 3.0 μL/mL, 4.0 μL/mL, and 5.0 μL/mL), which were dissolved in PDA with 1% DMSO. Fungal cultures (7 days old) were inoculated onto 4 mm diameter disks and cultured at 28 °C until complete mycelial coverage of negative controls was achieved. Each concentration included five technical replicates to ensure statistical validity. MIC was determined as the lowest AcEO concentration that completely suppressed fungal growth after a 7-day incubation period.

SEM was used to examine the impacts of AcEO on the morphology of *A. tenuissima* YJ-XY-GSC1 and *A. alternata* YJ-XY-GSC3. Following treatment with various concentrations of AcEO (specifically 0 and 0.25 MIC), the mycelia of *A. tenuissima* YJ-XY-GSC1 and *A. alternata* YJ-XY-GSC3 were gathered. Mycelial samples were immobilized in 2.5% glutaraldehyde for 12 h at 4 °C. Post-fixation, they were washed three times in 0.01 M phosphate buffer (PBS, pH 7.4). Dehydration was performed through an ascending ethanol series (50–100%, 10 min per step). Critical point drying with liquid CO_2_ was applied, followed by sputter-coating with gold. Morphological analysis was conducted under 3.0 kV accelerating voltage using a SU8010 scanning electron microscope (Hitachi, Tokyo, Japan).

## 5. Conclusions

This study characterized 124 fungal strains associated with black spot in Yanbian pear cultivars in China. Phylogenetic analysis revealed the dominant presence of *A. tenuissima* (54.0%, *n* = 67) and *A. alternata* (46.0%, *n* = 57). Our results also indicated that AcEO exhibits potent inhibitory activity against *A. tenuissima* and *A. alternate*, achieving complete suppression of mycelial growth at an MIC of 5.0 μL/mL.

The high volatility, poor water solubility, and susceptibility to degradation limited the application of essential oils. Further studies should focus on the preparation of a nanoemulsion using AcEO to maximize the efficacy in controlling pathogens causing black spot on Pingguoli. Additionally, the chemical components of plant essential oils are highly diverse. The components that truly have antibacterial and antifungal effects may only be one or a few of them. Moreover, there may be antagonistic or synergistic effects among various components. Further studies should also focus on the mechanisms of action and on extending the application of AcEO and the active monomer substances. This study provides new insights into alternative natural gaseous fungicides for black spot management, which may help to reduce economic losses.

## 6. Patents

Fu, Y. A raw material steaming device for plant essential oil production, 2023 (Patent No. CN117778103B).

## Figures and Tables

**Table 1:** Regional distribution and phylogenetic affiliations of *Alternaria* spp. isolates collected from symptomatic Yanbian Pingguoli pear foliage exhibiting characteristic black spot symptoms in China.

Geographic Districts of Yanbian	Number of Fields	Number of *Alternaria* Isolates	
		*A. tenuissima*	*A. alternata*
Longjing County, Zhixin Town	1	1 (1) ^a^	4 (2)
Longjing County, Fruit Farm	1	0 (0)	6 (2)
Longjing County, Shanhe Town	1	1 (1)	11 (3)
Helong County, Bajiazi Town	1	4 (2)	9 (3)
Helong County, Xicheng Town	1	5 (2)	0 (0)
Helong County, Longcheng Town	1	3 (2)	2 (1)
Tumen County, Yueqing Town	1	14 (3)	1 (1)
Tumen County, Liangshui Town	1	4 (2)	0 (0)
Yanji City, Yilan Town	2	14 (4)	3 (1)
Yanji City, Chaoyangchuan Town	1	5 (2)	7 (3)
Yanji City, Xiaoying Town	1	5 (2)	1 (1)
Hunchun County, Yingan Town	3	11 (5)	13 (5)
Total	15	67 (26)	57 (22)
Ratio	–	54.0%	46.0%

^a^ The parenthetical values denote the corresponding *Alternaria* strains employed in pathogenicity tests.

**Table 2:** Disease incidence and disease index of detached leaves of Yanbian Pingguoli pear caused by 48 *Alternaria* isolates.

*Alternaria* Species	Isolate Number	Disease Incidence (%) [Mean ± Standard Deviation (Range)]	Disease Index [Mean ± Standard Deviation (Range)]
*A. tenuissima*	26	71.2 ± 13.5 (51.7–100.0) ^a^	57.3 ± 6.9 (30.5–50.4) ^a^ *
*A. alternata*	22	48.2 ± 8.6 (31.2–100.0) ^b^	19.8 ± 3.7 (18.2–29.5) ^b^ *

* The mean ± standard deviation (SD) values (a,b) were calculated from triplicate experiments using Alternaria isolates specific to each Yanbian Pingguoli pear cultivar. Symptom severity (DS) was quantified following a 14-day incubation period under controlled conditions (28 °C, 90% RH). Incidence rates and disease indices, labeled with superscript letters, demonstrated statistically distinct variations (*p* < 0.05). For disease incidence, single-factor variance analysis (ANOVA) with Dunnett-T3 multiple comparisons revealed significant differences, whereas disease index data were processed using an LSD post hoc test for multiple comparisons.

**Table 3:** GC-MS analysis characterizing the volatile profile of *Artemisia capillaris* essential oil.

No.	Compound	Retention Time (min)	Molecular Formula	CAS	Relative Content (>0.05%)
1	Bicyclo [3.1.0]hex-2-ene	9.68	C_10_H_16_	2867-05-2	0.13
2	(1R)-2,6,6-Trimethylbicyclo[3.1.1]hept-2-ene	9.99	C_10_H_16_	7785-70-8	1.46
3	α-Pinene	10.17	C_10_H_16_	80-56-8	0.95
4 *	**2,2-Dimethyl-3-methylenebicyclo[2.2.1]heptane**	**10.71**	**C_10_H_16_**	**5794-04-7**	**5.03**
5	1-Isopropyl-4-meth-ylenebicyclo[3.1.0]hexane	11.85	C_10_H_16_	3387-41-5	1.97
6	Myrcene	12.49	C_10_H_16_	123-35-3	1.79
7	1-Octen-3-ol	13.65	C_9_H_16_O	3391-86-4	1.31
8 *	**Eucalyptol**	**15.20**	**C_10_H_18_O**	**470-82-6**	**60.40**
9	Cis-3,7-Dimethyl-1,3,6-octatriene	15.32	C_10_H_16_	3338-55-4	1.03
10	Crithmene	15.75	C_10_H_16_	99-85-4	1.96
11	Isoterpinolene	16.56	C_10_H_16_	586-63-0	0.2
12	Isothujone	17.62	C_10_H_16_O	471-15-8	1.73
13	Thujone	18.06	C_10_H_16_O	546-80-5	0.46
14	(4E,6Z)-2,6-Dimethyl-2,4,6-octatriene	18.43	C_10_H_16_	7216-56-0	0.84
15 *	**(-)-Alcanfor**	**19.30**	**C_10_H_16_O**	**76-22-2**	**3.86**
16	L(-)-Borneol	20.76	C_10_H_18_O	464-45-9	2.42
17	Terpinen-4-ol	20.89	C_10_H_18_O	562-74-3	0.75
18	Terpineol	21.33	C_10_H_18_O	98-55-5	0.52
19	(Z)-piperitol	21.73	C_10_H_18_O	16721-38-3	0.07
20	Capillin	23.12	C_12_H_18_O	495-74-9	2.26
21	1,7,7-Trimethylbicyclo[2.2.1]hept-2-yl acetate	24.32	C_12_H_20_O_2_	92618-89-8	0.31
22	(1S,3S,5S)-1-Isopropyl-4-methylenebicyclo[3.1.0]hexan-3-yl acetate	24.52	C_12_H_20_O_2_	139757-62-3	0.42
23	γ-Elemene	25.93	C_15_H_24_	29873-99-2	0.08
24	α-copaene	27.48	C_15_H_24_	3856-25-5	0.09
25 *	**β-copaene**	**29.15**	**C_15_H_24_**	**18252-44-3**	**3.25**
26	(-)-Isogermacrene D	29.86	C_15_H_24_	317819-80-0	0.10
27	(-)-Caryophyllene	30.11	C_15_H_24_	87-44-5	0.14
28	α-caryophyllene	30.27	C_15_H_24_	6753-98-6	0.10
29	(-)-Germacrene D	31.25	C_15_H_24_	23986-74-5	1.64
30	1,2,3,4,4a,5,6,7-octahydro-4a,5-dimethyl-3-(1-methylethenyl)-1-Naphthalenol	31.35	C_15_H_24_O	61847-19-6	0.16
31	bicyclogermacrene	31.60	C_15_H_24_	24703-35-3	0.05
32	ε-Muurolene	31.75	C_15_H_24_	30021-46-6	0.07
33	δ-Cadinene	32.34	C_15_H_24_	483-76-1	0.39
34	α-Panasinsene	33.11	C_15_H_24_	56633-28-4	0.64
35	(+)-Phyllocladene	34.50	C_20_H_32_	469-86-3	1.27
36	Gitoxigenin	35.71	C_23_H_34_O_5_	545-26-6	0.21
37	Muurolol	36.29	C_15_H_26_O	19912-62-0	0.17
38	Neointermedeol	36.90	C_15_H_26_O	5945-72-2	0.53
39	cis-p-mentha-2,8-dien-1-ol	37.50	C_10_H_16_O	425394-92-9	0.14
40	Bi-1-cycloocten-1-yl	42.04	C_16_H_26_	61468-42-6	0.55

* The components with high content % have been highlighted.

## Data Availability

The original contributions presented in this study are included in the article and Supplementary Materials. Further inquiries can be directed to the corresponding authors.

## Supplementary Materials

The following supporting information can be downloaded at: https://www.mdpi.com/article/10.3390/plants14203146/s1, Table S1: *Alternaria* isolates used for phylogenetic analysis; Table S2: Structural formulas of various components in the essential oil from *Artemisia capillaris*.
