# Supplementary material for: Antifungal Activity of Artemisia capillaris Essential Oil Against Alternaria Species Causing Black Spot on Yanbian Pingguoli Pear in China
Source: Plants (Basel). 2025 Oct 13;14(20):3146. doi: 10.3390/plants14203146 (PMC12567038; doi:10.3390/plants14203146)
Supplement: Supplementary file 1 [file plants-14-03146-s001.zip › plants-3901960-supplementary.pdf]

Supplementary Table S1 *Alternaria* isolates used for phylogenetic analysis

Isolate code	GenBank accession no.		Molecular identification
	histone 3 gene	rDNA ITS region	
LJ-ZX-GX1	OQ116345	OQ000949	*A. alternata*
LJ-ZX-GX2	OQ116346	OQ000950	*A. alternata*
LJ-ZX-GX3	OQ116347	OQ000951	*A. alternata*
LJ-ZX-GX4	OQ116348	OQ000952	*A. tenuissima*
LJ-ZX-GX5	OQ116349	OQ000953	*A. alternata*
LJ-GS-WD1	OQ116350	OQ000954	*A. alternata*
LJ-GS-WD2	OQ116351	OQ000955	*A. alternata*
LJ-GS-WD3	OQ116352	OQ000956	*A. alternata*
LJ-GS-WD4	OQ116353	OQ000957	*A. alternata*
LJ-GS-WD5	OQ116354	OQ000958	*A. alternata*
LJ-GS-WD6	OQ116355	OQ000959	*A. alternata*
LJ-SH-HX1	OQ116356	OQ000960	*A. alternata*
LJ-SH-HX2	OQ116357	OQ000961	*A. alternata*
LJ-SH-HX3	OQ116358	OQ000962	*A. alternata*
LJ-SH-HX4	OQ116359	OQ000963	*A. tenuissima*
LJ-SH-HX5	OQ116360	OQ000964	*A. alternata*
LJ-SH-HX6	OQ116361	OQ000965	*A. tenuissima*
LJ-SH-HX7	OQ116362	OQ000966	*A. alternata*
LJ-SH-HX8	OQ116363	OQ000967	*A. alternata*
LJ-SH-HX9	OQ116364	OQ000968	*A. alternata*
LJ-SH-HX10	OQ116365	OQ000969	*A. alternata*
LJ-SH-HX11	OQ116366	OQ000970	*A. alternata*
LJ-SH-HX12	OQ116367	OQ000971	*A. alternata*
HL-BJZ-NG1	OQ116368	OQ000972	*A. alternata*
HL-BJZ-NG2	OQ116369	OQ000973	*A. alternata*
HL-BJZ-NG3	OQ116370	OQ000974	*A. alternata*
HL-BJZ-NG4	OQ116371	OQ000975	*A. alternata*
HL-BJZ-NG5	OQ116372	OQ000976	*A. alternata*
HL-BJZ-NG6	OQ116373	OQ000977	*A. alternata*
HL-BJZ-NG7	OQ116374	OQ000978	*A. alternata*
HL-BJZ-NG8	OQ116375	OQ000979	*A. tenuissima*
HL-BJZ-NG9	OQ116376	OQ000980	*A. tenuissima*
HL-BJZ-NG10	OQ116377	OQ000981	*A. alternata*
HL-BJZ-NG11	OQ116378	OQ000982	*A. alternata*
HL-BJZ-NG12	OQ116379	OQ000983	*A. tenuissima*
HL-BJZ-NG13	OQ116380	OQ000984	*A. tenuissima*
HL-XC-BGC1	OQ116381	OQ000985	*A. tenuissima*
HL-XC-BGC2	OQ116382	OQ000986	*A. tenuissima*
HL-XC-BGC3	OQ116383	OQ000987	*A. tenuissima*
HL-XC-BGC4	OQ116384	OQ000988	*A. tenuissima*
HL-XC-BGC5	OQ116385	OQ000989	*A. tenuissima*
HL-LC-TS1	OQ116386	OQ000990	*A. alternata*
HL-LC-TS2	OQ116387	OQ000991	*A. alternata*
HL-LC-TS3	OQ116388	OQ000992	*A. tenuissima*
HL-LC-TS4	OQ116389	OQ000993	*A. tenuissim*
HL-LC-TS5	OQ116390	OQ000994	*A. tenuissima*
TM-YQ-WG1	OQ116391	OQ000995	*A. tenuissima*
TM-YQ-WG2	OQ116392	OQ000996	*A. tenuissima*
TM-YQ-WG3	OQ116393	OQ000997	*A. tenuissima*
TM-YQ-WG4	OQ116394	OQ000998	*A. tenuissima*
TM-YQ-WG5	OQ116395	OQ000999	*A. tenuissima*
TM-YQ-WG6	OQ116396	OQ001000	*A. tenuissima*
TM-YQ-WG7	OQ116397	OQ001001	*A. tenuissima*
TM-YQ-WG8	OQ116398	OQ001002	*A. tenuissima*
TM-YQ-WG9	OQ116399	OQ001003	*A. alternata*
TM-YQ-WG10	OQ116400	OQ001004	*A. tenuissima*
TM-YQ-WG11	OQ116401	OQ001005	*A. tenuissima*
TM-YQ-WG12	OQ116402	OQ001006	*A. tenuissima*

TM-YQ-WG13	OQ116403	OQ001007	*A. tenuissima*
TM-YQ-WG14	OQ116404	OQ001008	*A. tenuissima*
TM-YQ-WG15	OQ116405	OQ001009	*A. tenuissima*
TM-LS-ST1	OQ116406	OQ001010	*A. tenuissima*
TM-LS-ST2	OQ116407	OQ001011	*A. tenuissima*
TM-LS-ST3	OQ116408	OQ001012	*A. tenuissima*
TM-LS-ST4	OQ116409	OQ001013	*A. tenuissima*
YJ-YL-TY1	OQ116410	OQ001014	*A. alternata*
YJ-YL-TY2	OQ116411	OQ001015	*A. alternata*
YJ-YL-TY3	OQ116412	OQ001016	*A. alternata*
YJ-YL-TY4	OQ116413	OQ001017	*A. alternata*
YJ-YL-TY5	OQ116414	OQ001018	*A. tenuissima*
HC-SX-ED1	OQ116415	OQ001019	*A. tenuissima*
HC-SX-ED2	OQ116416	OQ001020	*A. alternata*
HC-SX-ED3	OQ116417	OQ001021	*A. tenuissima*
HC-SX-ED4	OQ116418	OQ001022	*A. tenuissima*
HC-SX-ED5	OQ116419	OQ001023	*A. tenuissima*
HC-SX-ED6	OQ116420	OQ001024	*A. alternata*
HC-SX-ED7	OQ116421	OQ001025	*A. alternata*
HC-SX-ED8	OQ116422	OQ001026	*A. tenuissima*
HC-SX-ED9	OQ116423	OQ001027	*A. alternata*
HC-SX-ED10	OQ116424	OQ001028	*A. alternata*
HC-SX-ED11	OQ116425	OQ001029	*A. alternata*
HC-SX-ED12	OQ116426	OQ001030	*A. alternata*
HC-SX-ED13	OQ116427	OQ001031	*A. alternata*
HC-SX-ED14	OQ116428	OQ001032	*A. alternata*
HC-HS-YD1	OQ116429	OQ001033	*A. alternata*
HC-HS-YD2	OQ116430	OQ001034	*A. alternata*
HC-HS-YD3	OQ116431	OQ001035	*A. alternata*
HC-HS-YD4	OQ116432	OQ001036	*A. alternata*
HC-HS-YD5	OQ116433	OQ001037	*A. tenuissima*
HC-SX-YD1	OQ116434	OQ001038	*A. alternata*
HC-SX-YD2	OQ116435	OQ001039	*A. tenuissima*
HC-SX-YD3	OQ116436	OQ001040	*A. alternata*
HC-SX-YD4	OQ116437	OQ001041	*A. alternata*
HC-SX-YD5	OQ116438	OQ001042	*A. tenuissima*
YJ-CYC-HC1	OQ116439	OQ001043	*A. tenuissima*
YJ-CYC-HC2	OQ116440	OQ001044	*A. alternata*
YJ-CYC-HC3	OQ116441	OQ001045	*A. alternata*
YJ-CYC-HC4	OQ116442	OQ001046	*A. alternata*
YJ-CYC-HC5	OQ116443	OQ001047	*A. alternata*
YJ-CYC-HC6	OQ116444	OQ001048	*A. alternata*
YJ-CYC-HC7	OQ116445	OQ001049	*A. alternata*
YJ-CYC-HC8	OQ116446	OQ001050	*A. alternata*
YJ-CYC-HC9	OQ116447	OQ001051	*A. tenuissima*
YJ-CYC-HC10	OQ116448	OQ001052	*A. alternata*
YJ-CYC-HC11	OQ116449	OQ001053	*A. tenuissima*
YJ-CYC-HC12	OQ116450	OQ001054	*A. tenuissima*
YJ-YL-LY1	OQ116451	OQ001055	*A. alternata*
YJ-YL-LY2	OQ116452	OQ001056	*A. alternata*
YJ-YL-LY3	OQ116453	OQ001057	*A. tenuissima*
YJ-YL-LY4	OQ116454	OQ001058	*A. tenuissima*
YJ-YL-LY5	OQ116455	OQ001059	*A. tenuissima*
YJ-YL-LY6	OQ116456	OQ001060	*A. tenuissima*
YJ-YL-LY7	OQ116457	OQ001061	*A. tenuissima*
YJ-YL-LY8	OQ116458	OQ001062	*A. tenuissima*
YJ-YL-LY9	OQ116459	OQ001063	*A. tenuissima*
YJ-YL-LY10	OQ116460	OQ001064	*A. alternata*
YJ-YL-LY11	OQ116461	OQ001065	*A. tenuissima*
YJ-YL-LY12	OQ116462	OQ001066	*A. tenuissima*
YJ-XY-GSC1	OQ116463	OQ001067	*A. tenuissima*
YJ-XY-GSC2	OQ116464	OQ001068	*A. tenuissima*
YJ-XY-GSC3	OQ116465	OQ001069	*A. alternata*
YJ-XY-GSC4	OQ116466	OQ001070	*A. tenuissima*
YJ-XY-GSC5	OQ116467	OQ001071	*A. tenuissima*
YJ-XY-GSC6	OQ116468	OQ001072	*A. tenuissima*

Supplementary Table S2 Structural formulas of various components in the essential oil from *Artemisia capillaris*

No.	Compound	Retention time (min)	Molecular formula	CAS	Structural formulas
1	Bicyclo[3.1.0]hex-2-ene	9.68	$C_{10}H_{16}$	2867-05-2	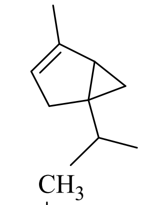
	(1R)-2,6,6-Trimethylbicyclo[3.1.1]hept-2-ene	9.99	$C_{10}H_{16}$	7785-70-8	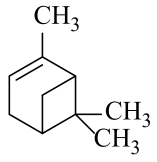
3	α-Pinene	10.17	$C_{10}H_{16}$	80-56-8	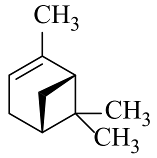
**4**	**2,2-Dimethyl-3-methylenebicyclo[2.2.1]heptane**	**10.71**	$\mathbf{C_{10}H_{16}}$	**5794-04-7**	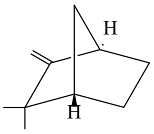
5	1-Isopropyl-4-meth-ylenebicyclo[3.1.0]hexane	11.85	$C_{10}H_{16}$	3387-41-5	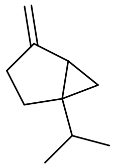
6	Myrcene	12.49	$C_{10}H_{16}$	123-35-3	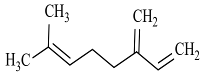
7	1-Octen-3-ol	13.65	$C_9H_{16}O$	3391-86-4	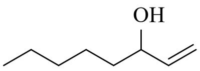
**8**	**Eucalyptol**	**15.20**	$\mathbf{C_{10}H_{18}O}$	**470-82-6**	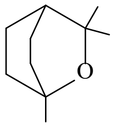
9	Cis-3,7-Dimethyl-1,3,6-octatriene	15.32	$C_{10}H_{16}$	3338-55-4	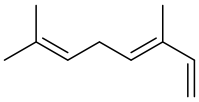
10	Crithmene	15.75	$C_{10}H_{16}$	99-85-4	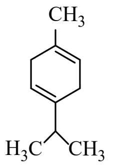
11	Isoterpinolene	16.56	$C_{10}H_{16}$	586-63-0	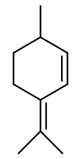
12	Isothujone	17.62	$C_{10}H_{16}O$	471-15-8	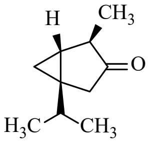

13	Thujone	18.06	$C_{10}H_{16}O$	546-80-5	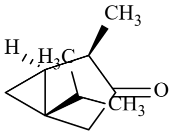
14	(4E,6Z)-2,6-Dimethyl-2,4,6-octatriene	18.43	$C_{10}H_{16}$	7216-56-0	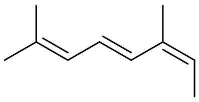
**15**	**(-)-Alcanfor**	**19.30**	**$C_{10}H_{16}O$**	**76-22-2**	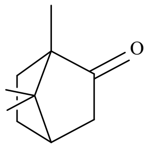
16	L(-)-Borneol	20.76	$C_{10}H_{18}O$	464-45-9	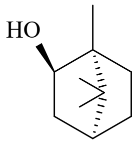
17	Terpinen-4-ol	20.89	$C_{10}H_{18}O$	562-74-3	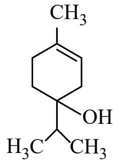
18	Terpineol	21.33	$C_{10}H_{18}O$	98-55-5	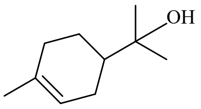
19	(Z)-piperitol	21.73	$C_{10}H_{18}O$	16721-38-3	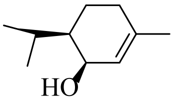
20	Capillin	23.12	$C_{12}H_{18}O$	495-74-9	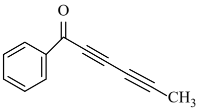
21	1,7,7-Trimethylbicyclo[2.2.1]hept-2-yl acetate	24.32	$C_{12}H_{20}O_2$	92618-89-8	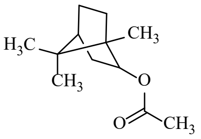
22	(1S,3S,5S)-1-Isopropyl-4-methylenebicyclo[3.1.0]hexan-3-yl acetate	24.52	$C_{12}H_{20}O_2$	139757-62-3	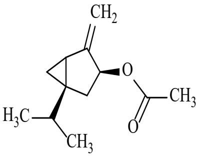
23	γ-Elemene	25.93	$C_{15}H_{24}$	29873-99-2	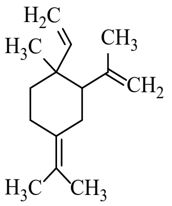
24	α-copaene	27.48	$C_{15}H_{24}$	3856-25-5	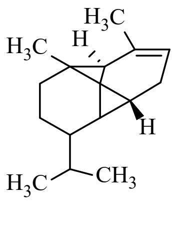

**25**	**β-copaene**	**29.15**	**$C_{15}H_{24}$**	**18252-44-3**	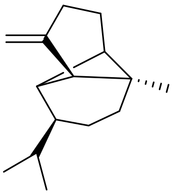
26	(-)-Isogermacrene D	29.86	$C_{15}H_{24}$	317819-80-0	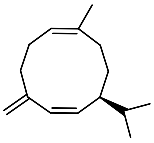
27	(-)-Caryophyllene	30.11	$C_{15}H_{24}$	87-44-5	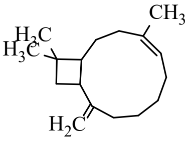
28	α-caryophyllene	30.27	$C_{15}H_{24}$	6753-98-6	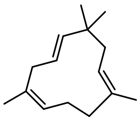
29	(-)-Germacrene D	31.25	$C_{15}H_{24}$	23986-74-5	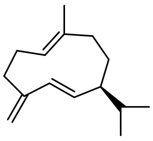
30	1-Naphthalenol, 1,2,3,4,4a,5,6,7-octahydro-4a,5-dimethyl-3-(1-methylethenyl)-	31.35	$C_{15}H_{24}O$	61847-19-6	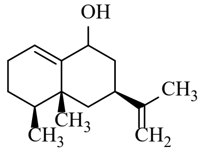
31	bicyclogermacrene	31.60	$C_{15}H_{24}$	24703-35-3	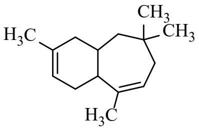
32	ε-Muurolene	31.75	$C_{15}H_{24}$	30021-46-6	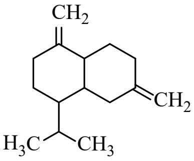
33	δ-Cadinene	32.34	$C_{15}H_{24}$	483-76-1	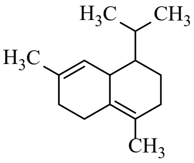
34	α-Panasinsene	33.11	$C_{15}H_{24}$	56633-28-4	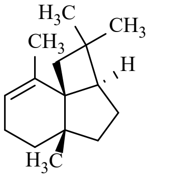
35	(+)-Phyllocladene	34.50	$C_{20}H_{32}$	469-86-3	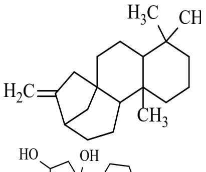
36	Gitoxigenin	35.71	$C_{23}H_{34}O_5$	545-26-6	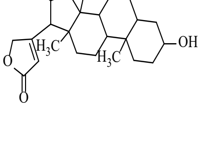

37	Muurolol	36.29	$C_{15}H_{26}O$	19912-62-0	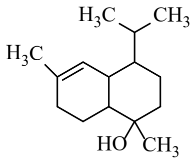
38	Neointermedeol	36.90	$C_{15}H_{26}O$	5945-72-2	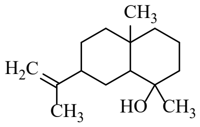
39	cis-p-mentha-2,8-dien-1-ol	37.50	$C_{10}H_{16}O$	425394-92-9	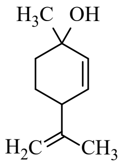
40	Bi-1-cycloocten-1-yl	42.04	$C_{16}H_{26}$	61468-42-6	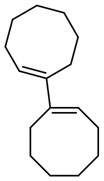
